# Annotations

**Published:** 1898-08-06

**Authors:** 


### ANNOTATIONS.

### The Causes of Lamp Accidents.

As a good deal which has lately been written about lamp accidents seems to take for granted that "explosion" is their common cause, and that their occurrence can be prevented by legislation directed to the raising of the "flash point," it may be well to point out once more that the low flash of an oil does not in itself cause or even predispose to the explosion of a lamp in which it is burned; indeed, the fact is probably just the other way. What the low flash does is to make the spilling of the oil, from whatever cause it may occur, a serious matter in many cases in which with an oil of a high flash point it might lead to but little mischief. When a lamp explodes, whether the oil be high flash or low flash, the oil itself will certainly be lighted. But accidents do not commonly arise from explosion; their common cause is the breaking of a fragile reservoir, and the scattering of the oil, and under these circumstances it depends to a large extent on the temperature at which inflammable vapour is given off whether a conflagration follows or not. When a lamp has been burning some time, the oil in the reservoir is often so hot that, even if of a high flash point, it would give off inflammable vapour; so that it must not be imagined for a moment that, so long as the present style of lamp is allowed to be sold, raising the flash point will put a stop to lamp accidents. But the amount of the vapour given off under such circumstances by a low is infinitely greater than by the high flash oil, and so is the probability of fire being communicated to it, and that is the real source of the mischief. It is the flame-carrying power of the large mass of vapour immediately given off by low flash oil, by aid of which slight accidents are turned into large ones, which is the real cause of the danger attending its use, not the supposed increased tendency to explosion, which indeed is very problematical.

### Infectious Diseases in General Hospitals.

A little quarrel at Perth, between the medical officer of health to the County Council and the staff of the Perth Royal Infirmary, brings to light a condition of affairs which we cannot regard as being in accordance with modern lights in regard to the treatment of scarlet fever. It appears that an arrangement has recently been in operation between the managers of the Royal Infirmary and the local authorities, whereby a certain number of beds were to be available for the reception of infectious cases sent by the latter. Meanwhile, the pressure on the Royal Infirmary having become considerable, it has been proposed that the managers should build a branch hospital for convalescents at Hillend, where the County Council also has its own hospital. Under these circumstances the medical officer to the County Council, with the object, apparently of bringing to an end this double arrangement, has indulged in certain criticisms of the present plan, which have brought back a protest from the staff of the Royal Infirmary. Into these matters we do not wish to enter. What, however, we do think should be cleared up, and what the persistence of such an arrangement as exists at Perth ought to give good facilities for clearing up, is the question, What is the practical result of treating scarlet fever cases in a general hospital? This is a matter which is of interest far beyond Perth. It appears that the infirmary there is a mixed one. The number of beds is given as 110, and the number of in-patients as 803 in the year. From the report before us we gather that 127 cases of scarlet fever were treated there in the year 1897, and that at times the actual number of patients suffering from that disease in the infirmary exceeded 40 at a time; so that at times more than a third of all the beds must have been occupied with scarlet fever cases, which, indeed, formed nearly a sixth of the whole number treated during the year. This hospital is returned as having one resident medical officer, one matron, six nurses, and five probationers, and it would be extremely interesting to hospital managers all over the country to know to what extent this large introduction of infectious disease affects the other patients, and what regulations are in force to prevent the spread of infection from one part of the house to another. On every hand all over the country money is being spent on the hypothesis that infectious diseases should not be mixed with others, or even among themselves. Either Perth is far behind the times, and is exposing its ordinary patients to unjustifiable risks, or the times are wrong, and a great deal of unnecessary expenditure is being undertaken in many other places.

### An American Comment on London Gynæcology.

The comments of strangers, when made honestly and as the result of personal observation, are always interesting and sometimes instructive. Dr. Preston Miller, of Washington, who has lately been contributing an admirable series of letters to the New York *Medical Record*, describing the surgical and gynæcological clinics which he visited during a tour in Europe, came to London on his way home, and apparently stayed only a short time. Among other things he notes are the comparatively infrequent use of the Paquelin cautery and the preference shown by all operators in London for the knife; the "greater liberty taken with the endometrium than at clinics in other cities, in the use of sounds, caustics, &c."; the old wooden operating-table, which admits of no Trendelenburg position, in one of our largest hospitals; the open fireplaces in operating rooms, "which are prolific of more smoke than heat"; the fact that the operating-room at Guy's has a floor of wide boards of soft wood, and contains "considerable furniture" and pictures, &c., on the walls; and the prevalence of ether as an anæsthetic. Perhaps, however, active and thoroughly wide-awake as Dr. Miller evidently has been in his travels, he did not see everything, for, he says, "the surgeons in London, like those of Paris, wear long finger nails, and, like their French neighbours, operate in chillingly cold rooms. Except for the smoke, I could have been comfortable in the London operating rooms with my overcoat on." Among his various remarks on Guy's Hospital we find the following comment on the gynæcologists. After describing how one of them, in treating fibroids, if necessary establishes premature menopause, leaving the tumour alone, he goes on to say, "His colleague takes the opposite view, preferring to remove the neoplasm and leave the ovaries in the pelvis, that the breasts may remain plump, the limbs round, the abdomen not take on fat, and the face not lose its feminine charm." An æsthetic reason for the choice of operation which evidently quite meets with his approval.

